# Associations between within-day step accumulation pattern and clinical measures of physical function: a change-for-change analysis of longitudinal data in community-dwelling older adults

**DOI:** 10.1186/s12966-025-01797-6

**Published:** 2025-07-15

**Authors:** Melvyn Hillsdon, Alexander Schoenfelder, Brad Metcalf, Afroditi Stathi, Max J. Western, Joss Langford

**Affiliations:** 1 Department of Public Health and Sport Sciences, University of Exeter, Exeter, EX1 2LU UK; 2 University of Exeter, Exeter, EX4 4QJ UK; 3 University of Birmingham, School of Sport, Exercise and Rehabilitation Sciences, University of Birmingham, Birmingham, B15 2TT UK; 4 Centre for Motivation and Behaviour change, Department for Health, University of Bath, Bath, BA2 7AY UK; 5 Activinsights Ltd, Huntingdon, PE28 0NJ UK

**Keywords:** Walking, Stepping, Physical function, Ageing

## Abstract

**Background:**

While daily step count and stepping pace are linked to various health benefits in older adults, less is known about how the pattern of step accumulation affects physical function. For example, the same step count could be accumulated through clusters of frequent, short bouts (e.g., during house cleaning) or fewer, longer bouts (e.g., walking to and from work). This study aimed to explore whether stepping patterns, and trends in these patterns, were associated with physical function in older adults.

**Methods:**

We analysed accelerometer data from wrist-worn GENEActiv devices, from four time points over 24 months in *n* = 597 older adults (age ≥65 years, 68% female) participating in the REtirement in ACTion intervention. A step counting algorithm was used to create bouts of stepping (at least 10 steps > 20 steps/minute) before counting the steps in each bout and the average cadence. Total daily steps (20–175 steps/minute), slower-paced steps (20–62 steps/minute; below the median cadence), and faster-paced walking steps (63–175 steps/minute; above the median cadence) were then calculated. We used the frequency of stepping bouts, the time between them (mean and standard deviation) and their burstiness (short bursts of stepping bouts clustered together), to examine the daily patterns of step accumulation. Linear mixed-effects models were used to assess trends in stepping variables and their association with changes in objectively measured physical function (short physical performance battery: SPPB) over the two-year period.

**Results:**

Total, slower-paced and faster-paced daily steps declined, along with the average number of stepping bouts. The time between stepping bouts increased. All components of burstiness, but not burstiness itself, were associated with changes in physical function, even when faster and slower steps (total steps) were in the same model (fewer stepping bouts = lower SPPB, greater SD = lower SPPB). Mean time between bouts was the strongest independent predictor, whereby a 10-minute increase in time between bouts was associated with a clinically important 0.46 decline in SPPB score (*p* < 0.001).

**Conclusions:**

Preventing increases in the time between stepping bouts could help preserve physical function in older adults. Future intervention trials targeting how bouts of stepping are spread throughout the day, rather than just total steps, may provide a more effective approach to promoting healthy physical functioning in older age.

**Supplementary Information:**

The online version contains supplementary material available at 10.1186/s12966-025-01797-6.

## Background

A higher number of daily steps (stepping volume) is linked to reduced all-cause and cause-specific mortality, as well as decreased risk of chronic diseases, including cardiovascular disease [1]. Both higher stepping volume and rate (the cadence of step accumulation, e.g., 62 steps/minute) have been jointly associated with lower rates of hospitalisation and all-cause mortality in older adults [2]. In addition, a higher stepping rate is associated with reduced all-cause, cancer, and cardiovascular morbidity and mortality, even when controlling for daily stepping volume [3]. Higher stepping volume and rate have also been associated with reduced health care costs [4]. Changes in the proportion of total daily steps undertaken at a higher rate have been associated with positive changes in objectively measured physical function, an important marker for quality of later life [5].

The volume and rate of stepping are not the only aspects that may affect physical function. In a cross-sectional study of 4378 middle aged adults from the 1970 British Cohort Study, patterns of step accumulation varied considerably by a variety of demographic characteristics [6]. In the cross-sectional Maastricht Study of 6085 adults, even after controlling for stepping volume, a higher number of stepping bouts was associated with poorer physical function [7]. Additionally, a pattern of upright postures, characterised by bursts of stepping followed by longer periods of rest, was associated with worse perceptions of physical function [7]. Together these findings imply that both the quantity of steps and the pattern in which they are accumulated vary among population sub-groups and may be related to physical function. However, the cross-sectional design of the studies prevents any conclusions about the direction of the relationship and how changes in the measures might be associated with changes in physical function.

Although older age is associated with reductions in preferred walking speed, cadence and stride length [8], what is not known is how daily patterns of stepping change with age and how changes in stepping patterns are associated with physical function. We are not aware of any other study that has reported associations between the trajectories of change in stepping bouts at different stepping rates, the pattern in which stepping bouts were accumulated and the trajectories of physical function.

Therefore, the primary aim of this study was to examine changes in the patterns of stepping bouts (periods of consecutive stepping events) and the association with changes in physical function in a cohort of older adults with mobility limitations over a period of two years. In addition, we examined 2-year trends in seven stepping metrics, across four time points, as well as how well they tracked from baseline to 2-year follow up.

## Methods

### Study design

Participants were drawn from the REtirement in ACTion (REACT) study. The REACT study was a pragmatic, multi-centre, single-blind, two-arm randomised controlled trial with an internal pilot, including process and economic evaluations. It tested a community-based physical activity and behaviour maintenance programme aimed at preventing mobility-related disability in UK adults aged 65 + at high risk of functional decline (Short Physical Performance Battery score: SPPB = 4–9 inclusive [9, 10]). In addition to the SPPB score, participants were eligible for the study if they reported being able to walk across a room, had not had recent heart surgery and were able to attend the intervention sessions. The study received ethical approval from the National Health Service (NHS) South East Coast-Surrey Research Ethics Committee (15/LO/2082) and was registered under ISRCTN45627165. All participants provided written informed consent, which included permission for the use of anonymised data in future research endeavours. The comprehensive study protocol has been detailed in previous publications [10–12].

The study followed participants for a period of two years and asked them to wear a wrist-worn GENEActiv accelerometer for seven consecutive days at four time points: baseline, 6 months, 12 months and 24 months. At each of these time points, participants also underwent a laboratory assessment to evaluate their physical function (SPPB score) and other health metrics. The SPPB score can range from 0 to 12 and is a composite measure of three sub-scores that evaluate the standing balance, walking speed and ability to rise from a chair on a scale of 0–4 (“Could not complete the task” to “Completed the task with no signs of decline in physical function” [13]).

The GENEActiv accelerometer has demonstrated excellent intra-device and inter-device reliability in measuring acceleration [14]. Its clinical validity was further established within the REACT study, where it was used to develop a digital susceptibility/risk biomarker associated with low physical function in community-dwelling older adults. In addition, the open-source GENEAclassify R package’s analytical validity was found to be excellent for total step count, moderate for walking and faster-paced steps, and low for slower-paced steps. However, the lower accuracy for slower-paced steps likely reflects limitations of the reference device rather than the GENEActiv system itself [5].

### Data cleaning and aggregation

This study replicates the data cleaning process that is explained and justified in a related analysis of the REACT dataset [5]. In short, bouts with fewer than ten steps and cadences below 20 steps/minute and above 175 steps/minute were excluded. At least 10 steps are required to provide an accurate estimate of the stepping rate [15] and our step counting algorithm is only valid for stepping rates ≥ 20 steps/minute [5]. The bout-level stepping data were then aggregated into variables of average daily stepping. To be included in the analysis, a participant had to provide complete data from at least two periods during which they wore the accelerometer on at least six days per period for at least 18 h per day (*n* = 597). Data from days beyond the 7th day of recording were excluded.

Although some studies have shown that less days are required to provide an estimate of average daily time in moderate to vigorous physical activity (MVPA), because this study was focused on measures of patterns of accumulation, a higher number of hours per day and days of wear were necessary. Further, studies in older adults and those using the same wrist-worn device have recommended ≥ 5 days of valid wear [16].

For each recording period and participant, the means of the daily counts for total steps (20–175 steps/minute), slower-paced steps (20–62 steps/minute), and faster-paced walking steps (63–175 steps/minute) were calculated (see Table 1 for definitions). The median walking cadence across all participants (62 steps/minute) delineated slower-paced from faster-paced walking. Using the study-specific median, rather than a fixed step rate selected from the literature, helps to ensure that definitions are tailored, meaningful, and reflect the capacities of the study population. This is especially important when working with older adults who have mobility limitations that differ significantly from general or younger populations.

This study also derived new measures to describe the patterns of step acquisition in bouts, known as burstiness, representing the extent to which bouts occur in clusters rather than being evenly spaced out over time [17–20]. The daily monitoring interval, during which physical activity and step accumulation occurred, was calculated as the time between the day’s first active stepping event after 3:00 a.m. (typical earliest mid-sleep time [21]) and the last active stepping event before midnight on the same day. The active monitoring interval had to be at least 12 h long and only events within this interval were used to calculate burstiness. Only participants who provided at least six such days were included in the analysis.

The GENEAclassify step counting algorithm uses changepoint detection to create variable-length bouts of homogenous data between transitions before counting the steps in each bout. It is possible that multiple stepping events (defined as events with at least ten steps and a cadence > 20 steps/minute) or inactive events (defined as events without steps) occurred adjacent to each other. Consecutive stepping events were combined into a single stepping bout that contained the sum of the steps and durations of the previously separate events. Inactive events were treated accordingly, resulting in an alternating sequence of stepping and inactive bouts.

### Stepping and burstiness measures

The burstiness measure was implemented with a variant that is robust against finite-size effects that could otherwise lead to erroneous ceilings [18] when few stepping bouts occurred:

$$\:{A}_{n}\left(\frac{{\sigma\:}_{n}}{{\mu\:}_{n}}\right)=\frac{\sqrt{n+1}\left(\frac{{{\upsigma\:}}_{n}}{{{\upmu\:}}_{n}}\right)-\sqrt{\text{n}-1}}{\left(\sqrt{\text{n}+1}-2\right)\left(\frac{{{\upsigma\:}}_{n}}{{{\upmu\:}}_{n}}\right)+\sqrt{\text{n}-1}}$$

for $$\:0\:\le\:\:$$$$\:\left(\frac{{\sigma\:}_{n}}{{\mu\:}_{n}}\right)\:\le\:\:\sqrt{n-1}\:$$, where *n* denotes the number of stepping bouts, *σ* the standard deviation of the times between stepping bouts and *µ* the mean time between stepping bouts. The burstiness was only calculated for days with at least ten stepping bouts to ensure that the necessary conditions were met. We calculated the burstiness for each valid day and then averaged the daily burstiness scores for each visit separately for each participant.

In total, seven measures of daily step acquisition were calculated as described in Table 1.

**Table 1:** Measures of daily step acquisition

Stepping measure	Description
Total steps	The total of all steps in a day acquired in bouts with > = 10 steps, achieved at cadence between 20–175 steps/minute.
Slower-paced steps	The total of slower-paced steps (20–62 steps/minute) acquired in a day including slower-paced walking steps (45–62 steps/minute) and slower-paced non-walking steps (20–44 steps/minute).
Faster-paced walking steps	The total of faster-paced walking steps (63–175 steps/minute) acquired in a day.
Number of stepping bouts	The count of distinct bouts of stepping separated by periods of non-stepping in the active monitoring window.
Mean time between stepping bouts	The mean duration of the inactive bouts that separate the stepping bouts in the active monitoring window.
SD of times between stepping bouts	The standard deviation of the inactive bout durations separating the stepping bouts in the active monitoring window.
Burstiness of stepping bouts	The daily composite burstiness measure ranging between − 1 and 1, where negative values indicate the coefficient of variation (SD/mean) of the ‘time between stepping bouts’ is less than 1 and positive values are the result of a coefficient of variation greater than 1.

### Statistical analysis

#### Baseline characteristics of the study sample

Medians (IQR: 25th– 75th percentiles) were reported for all continuous variables (all stepping measures, SPPB score, age and active monitoring window) because they were a mix of normal, slightly positively skewed and strongly positively skewed distributions. Counts (%) were reported for each category for all categorical variables (Table 2).

**Table 2:** Baseline characteristics of the study participants that met the inclusion criteria for longitudinal analysis

Number of participants	548/597 (92%)^1^
Gender^2^	
Female	374 (68%)
Male	174 (32%)
Age at recruitment (years)^3^	77 (72–82)
Site^2^	
Bristol/Bath	223 (41%)
Birmingham	132 (24%)
Exeter	193 (35%)
IMD quintile^2^	
1/5 (Most deprived)	59 (11%)
2/5	103 (19%)
3/5	109 (20%)
4/5	121 (22%)
5/5 (Least deprived)	156 (28%)
Highest education^2^	
Some/All secondary	241 (44%)
Some college	147 (27%)
All college/Degree	160 (29%)
Comorbidities^2^	
None	459 (84%)
One or more	89 (16%)
General Health (perceived SF-36)^2^	
Very good/Excellent	82 (15%)
Good	272 ( 50%)
Fair/Poor	194 (35%)
Group Allocation^2^	
Control	250 (46%)
Intervention	298 (54%)
Stepping Measures^1^	
Total steps (x1000 per day)	5.81 (4.00–7.93)
Slower-paced steps (x1000 per day)	4.85 (3.18–6.73)
Faster-paced walking steps (x1000 per day)	0.62 (0.19–1.37)
Burstiness of stepping bouts	0.32 (0.28–0.37)
Number of stepping bouts (per day)	55.8 (44.2–64.8)
Mean time between stepping bouts (mins)	15.5 (12.6–20.6)
SD of time between stepping bouts (mins)	27.2 (22.1–33.3)
Active monitoring interval (hours/day)^1^	16.7 (16.1–17.2)
Physical function (SPPB score)^1^	9 (8–10)

^1^ 548 of the 597 participants that had data from at least 2-time points had valid baseline data

^2^ N (%), ^3^ Median (IQR: 25th − 75th percentile)

#### Trends in the stepping measures

Linear mixed-effects modelling was used to quantify the trends in the seven stepping measures over the 2-year period via the ‘mixed’ command in Stata 18. Each stepping measure was entered as the outcome variable with ‘visit’ entered as a categorical fixed effect (baseline, 6,12 and 24 months). The models reported the mean difference (with 95% CI) between the baseline and 24-month visits and the corresponding Z-statistic and p-value. For ease of interpretation, the changes were also expressed as a percentage and as Cohen’s *d* (Table 3). Cohen’s *d* (z) was derived from ‘repeated measures’ using a linear mixed-effects model with the visit entered as a random effect and the participant ID as the cluster-level. The z-statistic that corresponds to the ‘baseline vs. 24mth’ coefficient in the model was converted to a Cohen’s *d* by dividing it by the √n.

**Table 3:** Changes in free-living stepping measures over a 2-year period (from baseline to 24-month visit)

Variable	units [95%CI]	%	Cohen’s d (Z)	*p*
Total steps (x1000/day)	-1.31 (-1.63 to -1.00)	-21	-0.36 (-8.18)	< 0.001
Slower steps (x1000/day)	-0.98 (-1.25 to -0.71)	-19	-0.32 (-7.22)	< 0.001
Faster-paced walking steps (x1000/day)	-0.33 (-0.46 to -0.21)	-33	-0.23 (-5.19)	< 0.001
Burstiness of stepping bouts	-0.01 (-0.02 to -0.00)	n/a	-0.09 (-2.13)	0.030
No. of stepping bouts (per day)	-5.39 (-7.03 to -3.75)	-10	-0.29 (-6.44)	< 0.001
Mean time between stepping bouts (minutes)	4.20 (3.24 to 5.17)	+ 24	0.38 (8.54)	< 0.001
SD of time between stepping bouts (minutes)	5.59 (4.26 to 6.91)	+ 19	0.37 (8.26)	< 0.001

#### Tracking of stepping measures

Pearson’s *r* and Spearman’s *rho* correlation analyses were used to quantify the degree of tracking for all seven stepping measures, determining how well a measure at baseline predicted the same measure at the 24-month time point. The analysis was based on all participants that had valid data for all seven stepping measures for both visits (Table 4).

**Table 4:** Tracking of the stepping measures over a 2-year period (from baseline to 24-month visit)

	Correlation from baseline to 24 months	
Stepping measures	Pearson’s r	Spearman’s rho
Total steps (x1000/day)	0.77	0.76
Slower steps (x1000/day)	0.78	0.77
Faster-paced walking steps (x1000/day)	0.73	0.70
Burstiness of stepping bouts	0.61	0.58
No. of stepping bouts (per day)	0.80	0.79
Mean time between stepping bouts (mins)	0.67	0.70
SD of time between stepping bouts (mins)	0.65	0.68

Correlations were based on *n* = 415/597 participants who had valid data for at least baseline and 24 months. All *p* < 0.001

#### Associations between changes in stepping measures and changes in physical function

Linear mixed-effects modelling was used to quantify the independent associations between changes in the stepping variables and changes in physical function over the 2-year period. The measure of physical function (SPPB score) ranged from 0 to 12 with data at all 13 ordinal categories and approximated to a normal distribution with a slight skewness of -0.53. It was therefore treated as a continuous outcome variable rather than a categorical one.

The models were built in two stages. The first stage tested whether total steps were associated with physical function independently of the covariates; and, specifically, whether the model fit was better when total steps were categorised in two cadence bands (slower-paced steps, faster-paced walking steps, Table 5a). This was achieved by comparing the step count-related models (Models 2 and 3) with the covariate-only model (Model 1). The second stage tested whether the burstiness of stepping bouts– or any of the three components that were used in its calculation (number of stepping bouts, mean time between stepping bouts, the SD of time between stepping bouts) when entered separately, in pairs and all together– were associated with physical function independently of the total steps and the covariates. This was achieved by comparing the fit of the models related to the burstiness of stepping bouts (Models 4, 5, 6i-iii, 7i-iii; see Table 5b for model variables) with the best-fitting ‘covariate + step count’ model (Model 2 or 3).

Two statistics were used to compare the models. Firstly, the change in conditional R^2^, which reflects the increase in the percentage of variation in the SPPB score that is explained by the fixed and random effects (greater increase = greater improvement). Secondly, the change in Akaike’s Information Criteria (AIC), which reflects the change in model fit relative to the number of additional predictors to account for the increased risk of overfitting (greater decrease = greater improvement). For each of these models, the unstandardised beta coefficient (95%CI), Z-statistic and p-value were reported for each stepping measure that was added to the model.

The covariates for every model were: active monitoring window, age at recruitment, gender, data collection site, perceived general health (SF-36 score), presence of a comorbidity, indices of multiple deprivation quintile (IMD; small area measures of relative deprivation across each of the constituent nations of the United Kingdom [22]), highest education qualification, measurement phase and experimental group (see Table 2 for variable levels). Data from the control and intervention groups were analysed together because the effect that the intervention had on the SPPB score was accounted for by including the ‘visit by experimental group’ interaction in the model.

For all models, the participant ID was entered as a random effect to define the participant as the cluster and to allow the intercepts to vary per participant. In all models, except the covariate-only model (Model 1), the stepping measures of interest were also entered as a random effect to allow the regression slopes to vary per individual.

**Table 5a:** Association between changes in free-living step counts (total, slower-paced and faster-paced walking) and changes in lab-based measures of physical function over a 2-year period (four time points)

		Predictor coefficients		Model fit	
Model no.	Burstiness-related predictors	B coefficient [95%CI]	Z (p)	R^2^_C_ % [∆ from Model 1]	AIC [∆ from Model 1]
1	Potential covariates^#^	n/a	n/a	18.5 n/a	7937 n/a
Adding Total steps to Model 1					
2	Total steps (1000 steps/day)	0.115 (0.077 to 0.153)	5.97 (< 0.001)	21.4 [+ 2.9]	7904 [-33]
Adding the two components of Total steps (Slower-paced and Faster-paced walking steps) to Model 1					
3	Slower-paced steps (1000 steps/day)	0.055 (0.009 to 0.100)	2.36 (0.018)	24.4 [+ 5.9]	7882 [-55]
	Faster-paced walking steps (1000 steps/day)	0.551 (0.375 to 0.728)	6.12 (< 0.001)		
	Faster-paced walking steps [squared] (1000 steps/day)	-0.052 (-0.081 to -0.022)	-3.46 (0.001)		

# Active monitoring interval (hours/day), Age at recruitment, Gender, ‘Gender x Age’ interaction, Site (Bath, Birmingham, Exeter), Health (Very good/Excellent, Good, Fair/Poor), Comorbidities (None, One or more), Deprivation (IMD), Education (highest level), Visit (Baseline, 6, 12, 24 months), Experimental group (control, intervention), ‘Visit x Experimental group’ interaction

**Table 5b:** Association between changes in the burstiness of free-living stepping bouts and changes in lab-based measures of physical function over a 2-year period (four time points)

		Predictor coefficients		Model fit	
Model no.	Burstiness-related predictors	B coefficient [95%CI]	Z (p)	R^2^_C_ % [∆ from Model 3]	AIC [∆ from Model 3]
Adding the ‘burstiness of stepping bouts’ measure to Model 3					
4	Burstiness of stepping bouts	1.04 [-0.32 to 2.39]	1.50 (0.135)	24.6 [+ 0.2]	7887 [+ 5]
Adding all three burstiness-related components to Model 3 simultaneously					
5	No. of stepping bouts (per day)	0.006 [-0.009 to 0.021]	0.82 (0.414)	26.1 [+ 1.7]	7853 [-29]
	Mean time between stepping bouts (mins)	-0.060 [-0.096 to -0.024]	-3.25 (0.001)		
	SD of time between stepping bouts (mins)	0.014 [-0.009 to 0.036]	1.17 (0.241)		
Adding each pair of the three burstiness-related components to Model 3 separately					
6i	No. of stepping bouts (per day)	0.006 [-0.009 to 0.021]	0.72 (0.471)	26.0 [+ 1.6]	7909 [-27]
	Mean time between stepping bouts (mins)	-0.043 [-0.065 to -0.020]	-3.71 (< 0.001)		
6ii	No. of stepping bouts (per day)	0.016 [0.002 to 0.030]	2.26 (0.024)	25.6 [+ 1.2]	7884 [+ 2]
	SD of time between stepping bouts (mins)	-0.015 [-0.030 to -0.000]	-1.97 (0.049)		
6iii	Mean time between stepping bouts (mins)	-0.056 [-0.091 to -0.020]	-3.07 (0.002)	25.8 [+ 1.4]	7830 [-52]
	SD of time between stepping bouts (mins)	0.007 [-0.017 to 0.030]	0.58 (0.564)		
Adding each burstiness-related component to Model 3 separately					
7i	No. of stepping bouts (per day)	0.030 [0.019 to 0.040]	5.65 (< 0.001)	25.4 [+ 1.0]	8027 [+ 145]
7ii	Mean time between stepping bouts (mins)	-0.046 [-0.066 to -0.027]	-4.67 (< 0.001)	25.7 [+ 1.3]	7827 [-55]
7iii	SD of time between stepping bouts (mins)	-0.025 [-0.037 to -0.013]	-4.02 (< 0.001)	25.1 [+ 0.7]	7844 [-38]

B = unstandardised Beta, R^2^_C_ = Conditional R^2^ (the variance explained by the fixed and random effects: higher = better), AIC = Akaike’s Information Criteria (model fit statistic that penalises for additional predictors: lower = better)

## Results

### Baseline characteristics of the study sample

At baseline, the sample meeting the inclusion criteria for analysis (*n* = 548/597) had a median age of 77 years and included 68% females, 11% from the least deprived quintile and 16% living with at least one comorbidity (Table 2). A median of 56 daily stepping bouts were achieved per day with a median 5180 total steps and 620 faster-paced walking steps per day.

### Trends of the stepping measures

All seven stepping measures changed over the 2-year period and all in the direction expected in a cohort of older adults with mobility limitations (Table 3). Total steps fell by 1310 steps/day (-21%, D = -0.36), slower-paced steps by 980 steps/day (-19%, D = -0.32) and faster-paced walking steps by 330 steps/day (-33%, D = -0.23). While the burstiness of stepping bouts decreased slightly (D = -0.09), there were much greater changes in its three components. On average, the number of daily stepping bouts decreased by 5.4 bouts (-10%, D = -0.29), the mean time between stepping bouts increased by 4.2 min (+ 24%, D = 0.38) and the SD of time between stepping bouts increased by 5.6 min (+ 19%, D = 0.37).

### Tracking of the stepping measures

All seven stepping measures demonstrated a moderate to high level of tracking over the 2-year period (Table 4). Pearson’s *r* and Spearman’s *rho* ranged from *r* = 0.73 to 0.77 and *rho* = 0.70 to 0.78 for the step count measures. For the burstiness measures they ranged from *r* = 0.61 to 0.80 and *rho* = 0.58 to 0.79.

### Associations between changes in stepping measures and changes in physical function

The covariate-only model (Model 1) explained 18.5% of the variation in the SPPB score (R^2^_C_ = 18.5%, AIC = 7937) (Table 5a). Splitting total steps into two separate cadence-based categories and entering them simultaneously (as three variables: slower-paced steps, faster-paced walking steps and faster-paced walking steps squared) led to a greater improvement from Model 1 (Model 3: ∆R^2^_C_ = + 5.9%, ∆AIC = -55) than when entering total steps as a single variable (Model 2: ∆R^2^_C_ = + 2.9%, ∆AIC = -33).

Consequently, the eight models that included at least one burstiness-related variable (Models 4, 5, 6i-iii, 7i-iii) were compared to Model 3 to determine whether such measures were associated with physical function independently of total steps by cadence category and the covariates (Table 5b).

Overall, each of the three components of burstiness, though not burstiness itself, were associated with physical function independently of total steps by cadence category and the covariates. However, the mean time between stepping bouts tended to be a slightly stronger independent predictor than the other two components of burstiness– the number of stepping bouts and SD of times between them– when entered simultaneously or separately (Table 5b). Therefore, of the eight models that tested at least one burstiness-related measure, the stepping measure coefficients from Model 7ii were chosen for interpretation (Table 6); especially as this model produced the greatest decrease in the AIC (-55) when compared to Model 3.

There was a negative linear association between changes in the mean time between stepping bouts and changes in the physical function, showing a reduction of the SPPB score by -0.046 points for each additional minute of time between stepping bouts.

The relationship between changes in the number of faster-paced walking steps and changes in physical function was characterised by a positive curvilinear association, with a positive linear term and a negative quadratic term (i.e. the prediction line for the SPPB score is steeper at lower levels of the faster-paced walking steps scale than it is at higher levels). So, for example, if faster-paced walking were to decrease by 1500 steps/day from 2500 to 1000 steps/day, the SPPB score would be predicted to decrease by 0.51 points. Yet, if the same 1500steps/day decrease was from 5000 to 3500steps/day, the predicted decrease in SPPB would be 0.12 points.

A negative linear association was found between changes in the number of slower-paced steps and changes in physical function, whereby an increase of 1000 slower-paced steps/day was associated with an SPPB score decrease of -0.067. This is not as unintuitive as it may first seem. If a person decreases the number of faster-paced walking steps, it is likely that many of these steps are now done at a slower pace, thus increasing the number of slower-paced steps that they do.

**Table 6:** Association between changes in the ‘mean time between stepping bouts’ and changes in lab-based measures of physical function over a 2-year period (four time points): model 7ii from Table 5b. (N = 597, mean visits/person = 3.4, total observations = 2008)

Model terms	B coefficient [95%CI]	Z	*p*
Stepping measures			
Mean time between stepping bouts (mins)	-0.046 [-0.066 to -0.027]	-4.67	< 0.001
Slower-paced steps (1000 steps)	-0.067 [-0.119 to -0.015]	-2.51	0.012
Faster-paced walking steps (1000 steps)	0.516 [0.353 to 0.678]	6.23	< 0.001
Faster-paced walking steps [squared] (1000^2^ steps)	-0.051 [-0.077 to -0.024]	-3.76	< 0.001
Covariates			
Active monitoring interval (per day)	-0.008 [-0.099 to 0.083]	-0.17	0.868
Age (years)	-0.068 [-0.089 to -0.046]	-6.18	< 0.001
Gender			
Female	0	n/a	n/a
Male	1.844 [-0.989 to 4.678]	1.28	0.202
Age (years) by Gender			
Age for Female	0	n/a	n/a
Age for Males	-0.024 [-0.061 to 0.012]	-1.30	0.194
Site			
Bath	0	n/a	n/a
Birmingham	0.375 [0.074 to 0.676]	2.44	0.015
Exeter	0.154 [-0.111 to 0.419]	1.14	0.256
General Health			
Very good/Excellent	0	n/a	n/a
Good	-0.238 [-0.448 to -0.027]	-2.21	0.027
Fair/Poor	-0.715 [-0.958 to -0.472]	-5.77	< 0.001
Comorbidities			
None	0	n/a	n/a
>=1	0.194 [-0.128 to 0.517]	1.18	0.237
Deprivation			
IMD quintile 1: Most deprived	0	n/a	n/a
IMD quintile 2	-0.051 [-0.491 to 0.390]	-0.23	0.822
IMD quintile 3	0.073 [-0.373 to 0.519]	0.32	0.747
IMD quintile 4	0.012 [-0.444 to 0.469]	0.05	0.958
IMD quintile 5: Least deprived	0.225 [-0.229 to 0.678]	0.97	0.331
Education (highest level)			
Some/all secondary school	0	n/a	n/a
Some college	0.070 [-0.205 to 0.345]	0.50	0.619
Complete college/degree	0.142 [-0.132 to 0.416]	1.01	0.311
Visit			
Baseline	0	n/a	n/a
6 months	0.822 [0.574 to 1.070]	6.50	< 0.001
12 months	0.732 [0.480 to 0.984]	5.70	< 0.001
24 months	0.602 [0.343 to 0.861]	4.55	< 0.001
Experimental group			
Control at baseline	0	n/a	n/a
Intervention at baseline	0.141 [-0.148 to 0.429]	0.95	0.340
Visit by Experimental group			
Control at baseline	0	n/a	n/a
Control at 6 months	0	n/a	n/a
Control at 12 months	0	n/a	n/a
Control at 24 months	0	n/a	n/a
Intervention at baseline	0	n/a	n/a
Intervention at 6 months	0.626 [0.292 to 0.960]	3.67	< 0.001
Intervention at 12 Months	0.585 [0.247 to 0.924]	3.39	< 0.001
Intervention at 24 Months	0.538 [0.189 to 0.887]	3.02	0.002
Intercept	13.55 [11.32 to 15.79]	11.91	< 0.001

B = unstandardised Beta, IMD = Index of Multiple Deprivation

## Discussion

The primary aim of this study was to examine longer-term changes in patterns of daily stepping bouts and the association with changes in physical function independently of changes in stepping volume and rate. We are not aware of any other study that has reported associations between the trajectories of change in stepping bouts at different stepping rates, the pattern in which stepping bouts were accumulated, and the trajectories of physical function.

The burstiness of daily stepping, our main measure of the stepping pattern, was almost constant for the 2-year period of observation and was not associated with a decline in physical function when controlling for total steps. However, the burstiness measure is a composite of the number of stepping bouts per day, mean time between the stepping bouts and the standard deviation of the time between them– all three of which were associated with a decline in physical function, even when controlling for total steps.

The average time between stepping bouts was the burstiness-related component most strongly associated with a decline in physical function, through a negative linear relationship. A 10-minute increase in the mean time between stepping bouts was associated with a decrease of 0.46 points in the SPPB score– a similar magnitude of change in physical function as a 1000 steps/day decrease in faster-paced walking steps. Changes in the SPPB score of greater than 0.4 have been reported as a clinically important difference [23]. A 10-minute change in this measure is plausible: 13% of the participants decreased their mean time between stepping bouts by ≥10 min over the 2-year period.

In addition to changes in daily patterns of stepping, reductions in both slower-paced and faster-paced walking steps were associated with reductions in physical function. Depending on the level of faster-paced walking steps recorded at baseline, a reduction of 1000 or 1500 faster-paced walking steps/day was associated with a reduction in SPPB of ~ 0.50 units (e.g., if faster steps/day at baseline was 1000 and over time this decreased to 0, then the predicted decrease in SPPB would be -0.46 units over that same time. Yet, if faster-paced steps/day at baseline was 2500, this would need to decrease by 1500 steps - to 1000 - to predict a similar decrease in the SPPB score of -0.51 units).

Faster-paced stepping has been linked to a wide range of health outcomes including reduced all-cause, cancer, cardiometabolic risk factors and cardiovascular morbidity and mortality, independent of daily step count [3, 24]. In this study, we defined faster-paced walking as above the median stepping rate for the sample (62 steps/minute). This was lower than the much-cited 100 steps/minute recommended to meet current public health guidelines [25]. However, stepping at ≥ 100 steps/minute in everyday living is rare, with most stepping recorded at cadences < 60 steps/minute, consistent with the findings of this study [26]. In a sample of adults with a median age of 75 years, a cadence of approximately 70 steps/minute has been shown to be associated with moderate intensity physical activity, the intensity required to meet physical activity recommendations [27]. However, other studies have reported that higher cadences are required to achieve moderate intensity [25]. The likely explanation for these discrepancies is cohort differences between studies.

The changes in stepping volume, rate and clustering of stepping throughout the day are consistent with each other, indicating that the participants’ physical function suffered as they aged. During the observation period, total steps per day decreased, with a proportionally greater loss of faster-paced walking steps than slower-paced steps. This was accompanied by a reduction in the number of stepping bouts per day while the periods between stepping bouts became longer and their variability increased. Prolonged periods of sedentary behaviour have been associated with negative health outcomes in older adults [28] and increasing time spent per day in sedentary bouts longer than 30-minutes has also been associated with worsening quality of life in older adults [29]. The pattern of declining steps per day, especially at higher cadences, along with longer gaps between stepping bouts, could result from declining endurance capacity and a greater need for rest following exertion. Endurance capacity declines with age, especially after the age of 60 [30], so that a greater number of everyday activities, including walking, induce fatigue and muscular discomfort.

If declines in stepping bouts and longer times between them do reflect reduced endurance capacity or even pre-clinical disease, then the remote monitoring of trajectories in patterns of stepping in the community may be a useful indicator of a decline in function that may be observable before it would be detectable by laboratory measures of function. In addition, large-scale monitoring of stepping patterns is more practical than getting large numbers of older people into laboratories for performance measures.

### Strengths and limitations

Our study has several strengths. We used objective data of both exposure and outcome measures. The prospective design with multiple measures of exposure and outcome, in a large sample of older adults, allowed us to model prospective associations between changes in stepping behaviour and changes in physical function as participants aged. However, reverse causation cannot be ruled out. Further, the stepping measures were derived from the validated pfSTEP digital biomarker of the susceptibility/risk of declining physical function in community-dwelling older adults [5]. Finally, we have shown that the benefits of stepping pace and the pattern of accumulation are independent of each other and not explained by other important covariates.

There were also limitations to the study. Although our stepping measures were based on a validated digital biomarker, there is an ongoing challenge in estimating slower-paced stepping, especially in older adults [31–35]. Consequently, the number of slower-paced steps may have been underestimated.

Including participants with data from as few as two visits (*N* = 597) had the potential to introduce non-attendance (not participating in follow up appointments) and/or non-adherence (not wearing the accelerometer each day) bias, though the latter was mitigated by including only those with valid data from at least six of the seven days per visit. To test whether this potential source of bias affected the findings, a sensitivity analysis replicated the final model (Model 7ii) with only those participants that provided data for three or more visits (*n* = 510) and all four visits (*n* = 304). The association statistics for faster-paced walking steps (linear and quadratic) and for the mean time between stepping bouts from these two models were very similar to those derived from participants with data at two or more visits (Additional File 1). The association for slower-paced steps was weaker in the ‘all four visits’-model compared to the ‘two or more visits’-model, though this alone was not considered to be evidence of non-attendance and/or non-adherence bias. Our best model only explained 25.7% of the variance in SPPB change. However, this needs to be interpreted in the context of the limited resolution of the physical function measure, which is scored on a 12-point scale derived from just three very short lab-based tests. This may restrict the model’s capacity to explain more detailed variation and therefore the observed R^2^ = 0.257 is likely to be an under-estimate of the true shared variance. Notwithstanding, the observed increase in R^2^ due to the addition of the stepping metrics was 7.2%, which is considered a ‘moderate’ improvement.

## Conclusion

We have shown that preserving the number of stepping bouts, especially faster-paced ones, was associated with a slowing of decline in physical function in older adults. Further, decreasing the time between stepping bouts would be expected to help to protect levels of physical function to an even greater degree. Increasing the proportion of faster-paced stepping bouts, without necessarily increasing total steps, may lower the risk of low physical function in older adults. Trials of the effects of interventions that can help older adults to increase the proportion of faster-paced stepping bouts each day, while reducing the time between them, are needed to determine if such an approach would be more effective than simply promoting more steps per day. The stepping metrics reported in this paper could be used to identify people with declining physical function before they experience symptoms that require clinical testing.

## Electronic Supplementary Material

Below is the link to the electronic supplementary material.

Additional file 1

## Data Availability

The data presented in this study are available on request from Afroditi Stahti (REACT dataset).

## Abbreviations

SPPB: Short Physical Performance Battery
IMD: Index of Multiple Deprivation
REACT: REtirement in ACTion study

## References

[1] Hall KS, Hyde ET, Bassett DR, Carlson SA, Carnethon MR, Ekelund U et al. Systematic review of the prospective association of daily step counts with risk of mortality, cardiovascular disease, and dysglycemia. Int J Behav Nutr Phys Act [Internet]. 2020 Dec [cited 2022 Dec 7];17(1):78. Available from: https://ijbnpa.biomedcentral.com/articles/10.1186/s12966-020-00978-9 10.1186/s12966-020-00978-9 PMC7305604 32563261

[2] Mañas A, del Pozo Cruz B, Ekelund U, Losa Reyna J, Rodríguez Gómez I, Carnicero Carreño JA et al. Association of accelerometer-derived step volume and intensity with hospitalizations and mortality in older adults: A prospective cohort study. J Sport Health Sci [Internet]. 2022 Sep [cited 2022 Dec 5];11(5):578–85. Available from: https://linkinghub.elsevier.com/retrieve/pii/S2095254621000521 10.1016/j.jshs.2021.05.004 PMC9532608 34029758

[3] del Pozo Cruz B, Ahmadi MN, Lee IM, Stamatakis E. Prospective Associations of Daily Step Counts and Intensity With Cancer and Cardiovascular Disease Incidence and Mortality and All-Cause Mortality. JAMA Intern Med [Internet]. 2022 Nov 1 [cited 2022 Dec 5];182(11):1139. Available from: https://jamanetwork.com/journals/jamainternalmedicine/fullarticle/2796058 10.1001/jamainternmed.2022.4000 PMC9468953 36094529

[4] Wohlrab M, Klenk J, Delgado-Ortiz L, Chambers M, Rochester L, Zuchowski M et al. The value of walking: a systematic review on mobility and healthcare costs. Eur Rev Aging Phys Act [Internet]. 2022 Dec [cited 2024 Jul 28];19(1):31. Available from: https://eurapa.biomedcentral.com/articles/10.1186/s11556-022-00310-3 10.1186/s11556-022-00310-3 PMC9798720 36581809

[5] Schoenfelder A, Metcalf B, Langford J, Stathi A, Western MJ, Hillsdon M. The Analytical and Clinical Validity of the pfSTEP Digital Biomarker of the Susceptibility/Risk of Declining Physical Function in Community-Dwelling Older Adults. Sensors [Internet]. 2023 May 27 [cited 2024 Jul 28];23(11):5122. Available from: https://www.mdpi.com/1424-8220/23/11/5122 10.3390/s23115122 PMC10255880 37299849

[6] Culverhouse J, Hillsdon M, Pulsford R. Cross-sectional associations between temporal patterns and composition of upright and stepping events with physical function in midlife: Insights from the 1970 British Cohort Study. Scand J Med Sci Sports [Internet]. 2024 May [cited 2024 Jul 28];34(5):e14645. Available from: https://onlinelibrary.wiley.com/doi/10.1111/sms.14645 10.1111/sms.14645 38736180

[7] Culverhouse J, Hillsdon M, Koster A, Bosma H, De Galan BE, Savelberg HHCM et al. Cross-sectional associations between patterns and composition of upright and stepping events with physical function: insights from The Maastricht Study. Eur Rev Aging Phys Act [Internet]. 2024 May 9 [cited 2024 Jul 28];21(1):10. Available from: https://eurapa.biomedcentral.com/articles/10.1186/s11556-024-00343-w 10.1186/s11556-024-00343-w PMC11080173 38724917

[8] Herssens N, Verbecque E, Hallemans A, Vereeck L, Van Rompaey V, Saeys W. Do spatiotemporal parameters and gait variability differ across the lifespan of healthy adults? A systematic review. Gait Posture [Internet]. 2018 Jul [cited 2024 Jul 28];64:181–90. Available from: https://linkinghub.elsevier.com/retrieve/pii/S0966636218307094 10.1016/j.gaitpost.2018.06.012 29929161

[9] Stathi A, Greaves CJ, Thompson JL, Withall J, Ladlow P, Taylor G et al. Effect of a physical activity and behaviour maintenance programme on functional mobility decline in older adults: the REACT (Retirement in Action) randomised controlled trial. Lancet Public Health [Internet]. 2022 Apr [cited 2024 Nov 28];7(4):e316–26. Available from: https://linkinghub.elsevier.com/retrieve/pii/S2468266722000044 10.1016/S2468-2667(22)00004-4 PMC8967718 35325627

[10] Snowsill TM, Stathi A, Green C, Withall J, Greaves CJ, Thompson JL et al. Cost-effectiveness of a physical activity and behaviour maintenance programme on functional mobility decline in older adults: an economic evaluation of the REACT (Retirement in Action) trial. Lancet Public Health [Internet]. 2022 Apr [cited 2025 May 26];7(4):e327–34. Available from: https://linkinghub.elsevier.com/retrieve/pii/S2468266722000305 10.1016/S2468-2667(22)00030-5 PMC8967720 35325628

[11] Stathi A, Withall J, Greaves CJ, Thompson JL, Taylor G, Medina-Lara A et al. A community-based physical activity intervention to prevent mobility-related disability for retired older people (REtirement in ACTion (REACT)): study protocol for a randomised controlled trial. Trials [Internet]. 2018 Dec [cited 2022 Dec 5];19(1):228. Available from: https://trialsjournal.biomedcentral.com/articles/10.1186/s13063-018-2603-x 10.1186/s13063-018-2603-x PMC5905123 29665854

[12] Withall J, Greaves CJ, Thompson JL, de Koning JL, Bollen JC, Moorlock SJ et al. The Tribulations of Trials: Lessons Learnt Recruiting 777 Older Adults Into REtirement in ACTion (REACT), a Trial of a Community, Group-Based Active Aging Intervention Targeting Mobility Disability. Newman A, editor. J Gerontol Ser A [Internet]. 2020 Nov 13 [cited 2022 Dec 5];75(12):2387–95. Available from: https://academic.oup.com/biomedgerontology/article/75/12/2387/5799245 10.1093/gerona/glaa051 PMC7662171 32147709

[13] Guralnik JM, Simonsick EM, Ferrucci L, Glynn RJ, Berkman LF, Blazer DG et al. A Short Physical Performance Battery Assessing Lower Extremity Function: Association With Self-Reported Disability and Prediction of Mortality and Nursing Home Admission. J Gerontol [Internet]. 1994 Mar 1 [cited 2022 Jun 4];49(2):M85–94. Available from: https://academic.oup.com/geronj/article-lookup/doi/10.1093/geronj/49.2.M85 10.1093/geronj/49.2.m85 8126356

[14] Esliger DW, Rowlands AV, Hurst TL, Catt M, Murray P, Eston RG. Validation of the GENEA Accelerometer. Med Sci Sports Exerc [Internet]. 2011 Jun [cited 2022 Jun 4];43(6):1085–93. Available from: https://journals.lww.com/00005768-201106000-00022 10.1249/MSS.0b013e31820513be 21088628

[15] Toth LP, Park S, Pittman WL, Sarisaltik D, Hibbing PR, Morton AL et al. Effects of Brief Intermittent Walking Bouts on Step Count Accuracy of Wearable Devices. J Meas Phys Behav [Internet]. 2019 Mar [cited 2022 Jun 18];2(1):13–21. Available from: https://journals.humankinetics.com/doi/10.1123/jmpb.2018-0050

[16] Hart TL, Swartz AM, Cashin SE, Strath SJ. How many days of monitoring predict physical activity and sedentary behaviour in older adults? Int J Behav Nutr Phys Act [Internet]. 2011 Dec [cited 2025 May 26];8(1):62. Available from: https://ijbnpa.biomedcentral.com/articles/10.1186/1479-5868-8-62 10.1186/1479-5868-8-62 PMC3130631 21679426

[17] Goh KI, Barabási AL. Burstiness and memory in complex systems. EPL Europhys Lett [Internet]. 2008 Feb [cited 2024 Jul 22];81(4):48002. Available from: https://iopscience.iop.org/article/10.1209/0295-5075/81/48002

[18] Kim EK, Jo HH. Measuring burstiness for finite event sequences. Phys Rev E [Internet]. 2016 Sep 15 [cited 2024 Jul 14];94(3):032311. Available from: http://arxiv.org/abs/1604.01125 10.1103/PhysRevE.94.032311 27739800

[19] Berardi V, Pincus D, Walker E, Adams MA. Burstiness and Stochasticity in the Malleability of Physical Activity. J Sport Exerc Psychol [Internet]. 2021 Oct 1 [cited 2024 Jul 14];43(5):387–98. Available from: https://journals.humankinetics.com/view/journals/jsep/43/5/article-p387.xml 10.1123/jsep.2020-0340 PMC9792373 34504039

[20] Takeuchi M, Sano Y. Burstiness of human physical activities and their characterization [Internet]. arXiv; 2022 [cited 2024 Jul 14]. Available from: http://arxiv.org/abs/2204.00201

[21] Roenneberg T, Kuehnle T, Juda M, Kantermann T, Allebrandt K, Gordijn M et al. Epidemiology of the human circadian clock. Sleep Med Rev [Internet]. 2007 Dec [cited 2025 Feb 5];11(6):429–38. Available from: https://linkinghub.elsevier.com/retrieve/pii/S1087079207000895 10.1016/j.smrv.2007.07.005 17936039

[22] McLennan D, Noble S, Noble M, Plunkett E, Wright G, Gutacker N. English Indices of Deprivation 2019 [Internet]. London: Ministry of Housing, Communities and Local Government; 2019 Sep [cited 2025 May 26] p. 117. Available from: https://assets.publishing.service.gov.uk/media/5d8b387740f0b609909b5908/IoD2019_Technical_Report.pdf

[23] Kwon S, Perera S, Pahor M, Katula JA, King AC, Groessl EJ et al. What is a meaningful change in physical performance? Findings from a clinical trial in older adults (the LIFE-P study). J Nutr Health Aging [Internet]. 2009 Jun [cited 2023 Feb 16];13(6):538–44. Available from: http://link.springer.com/10.1007/s12603-009-0104-z 10.1007/s12603-009-0104-z PMC3100159 19536422

[24] Garduno AC, LaCroix AZ, LaMonte MJ, Dunstan DW, Evenson KR, Wang G et al. Associations of Daily Steps and Step Intensity With Incident Diabetes in a Prospective Cohort Study of Older Women: The OPACH Study. Diabetes Care [Internet]. 2022 Feb 1 [cited 2022 Dec 6];45(2):339–47. Available from: https://diabetesjournals.org/care/article/45/2/339/139189/Associations-of-Daily-Steps-and-Step-Intensity 10.2337/dc21-1202 PMC8914434 35050362

[25] Tudor-Locke C, Han H, Aguiar EJ, Barreira TV, Schuna JM Jr, Kang M et al. How fast is fast enough? Walking cadence (steps/min) as a practical estimate of intensity in adults: a narrative review. Br J Sports Med [Internet]. 2018 Jun [cited 2022 Aug 5];52(12):776–88. Available from: https://bjsm.bmj.com/lookup/doi/10.1136/bjsports-2017-097628 10.1136/bjsports-2017-097628 PMC6029645 29858465

[26] Tudor-Locke C, Camhi SM, Leonardi C, Johnson WD, Katzmarzyk PT, Earnest CP et al. Patterns of adult stepping cadence in the 2005–2006 NHANES. Prev Med [Internet]. 2011 Sep [cited 2022 Jun 4];53(3):178–81. Available from: https://linkinghub.elsevier.com/retrieve/pii/S0091743511002301 10.1016/j.ypmed.2011.06.004 21708187

[27] Yates T, Henson J, McBride P, Maylor B, Herring LY, Sargeant JA et al. Moderate-intensity stepping in older adults: insights from treadmill walking and daily living. Int J Behav Nutr Phys Act [Internet]. 2023 Mar 18 [cited 2024 Jul 14];20(1):31. Available from: https://ijbnpa.biomedcentral.com/articles/10.1186/s12966-023-01429-x 10.1186/s12966-023-01429-x PMC10024004 36934275

[28] Wullems JA, Verschueren SMP, Degens H, Morse CI, Onambélé GL. A review of the assessment and prevalence of sedentarism in older adults, its physiology/health impact and non-exercise mobility counter-measures. Biogerontology [Internet]. 2016 Jun [cited 2024 Oct 20];17(3):547–65. Available from: http://link.springer.com/10.1007/s10522-016-9640-1 10.1007/s10522-016-9640-1 PMC4889631 26972899

[29] Yerrakalva D, Hajna S, Suhrcke M, Wijndaele K, Westgate K, Khaw KT et al. Associations between change in physical activity and sedentary time and health-related quality of life in older english adults: the EPIC-Norfolk cohort study. Health Qual Life Outcomes [Internet]. 2023 Jun 22 [cited 2024 Oct 20];21(1):60. Available from: https://hqlo.biomedcentral.com/articles/10.1186/s12955-023-02137-7 10.1186/s12955-023-02137-7 PMC10288723 37349799

[30] Mendonca GV, Pezarat-Correia P, Vaz JR, Silva L, Heffernan KS. Impact of Aging on Endurance and Neuromuscular Physical Performance: The Role of Vascular Senescence. Sports Med [Internet]. 2017 Apr [cited 2024 Oct 27];47(4):583–98. Available from: http://link.springer.com/10.1007/s40279-016-0596-8 10.1007/s40279-016-0596-8 27459861

[31] Lee KY, Macfarlane DJ, Cerin E. Comparison of three models of actigraph accelerometers during free living and controlled laboratory conditions. Eur J Sport Sci [Internet]. 2013 May [cited 2022 Aug 20];13(3):332–9. Available from: http://www.tandfonline.com/doi/abs/10.1080/17461391.2011.643925 10.1080/17461391.2011.643925 23679150

[32] Stansfield B, Hajarnis M, Sudarshan R. Characteristics of very slow stepping in healthy adults and validity of the activPAL3^™^ activity monitor in detecting these steps. Med Eng Phys [Internet]. 2015 Jan [cited 2022 Jun 4];37(1):42–7. Available from: https://linkinghub.elsevier.com/retrieve/pii/S1350453314002549 10.1016/j.medengphy.2014.10.003 25455167

[33] Sellers C, Dall P, Grant M, Stansfield B. Validity and reliability of the activPAL3 for measuring posture and stepping in adults and young people. Gait Posture [Internet]. 2016 Jan [cited 2022 Aug 7];43:42–7. Available from: https://linkinghub.elsevier.com/retrieve/pii/S0966636215009297 10.1016/j.gaitpost.2015.10.020 26669950

[34] John D, Morton A, Arguello D, Lyden K, Bassett D. What Is a Step? Differences in How a Step Is Detected among Three Popular Activity Monitors That Have Impacted Physical Activity Research. Sensors [Internet]. 2018 Apr 15 [cited 2022 Aug 9];18(4):1206. Available from: http://www.mdpi.com/1424-8220/18/4/1206 10.3390/s18041206 PMC5948774 29662048

[35] Bourke AK, Ihlen EAF, Helbostad JL. Validation of the activPAL3 in Free-Living and Laboratory Scenarios for the Measurement of Physical Activity, Stepping, and Transitions in Older Adults. J Meas Phys Behav [Internet]. 2019 Jun [cited 2022 Jun 18];2(2):58–65. Available from: https://journals.humankinetics.com/doi/10.1123/jmpb.2018-0056

