# Supplementary material for: Associations between within-day step accumulation pattern and clinical measures of physical function: a change-for-change analysis of longitudinal data in community-dwelling older adults
Source: Int J Behav Nutr Phys Act. 2025 Jul 15;22:98. doi: 10.1186/s12966-025-01797-6 (PMC12261632; doi:10.1186/s12966-025-01797-6)
Supplement: Supplementary file 1 — Additional file 1 [file 12966_2025_1797_MOESM1_ESM.docx]

**Additional Files**

**Additional File 1.** **Sensitivity analysis for the impact of requiring data from two, three or four visits on the association of the two key stepping measures – faster-paced walking steps (linear and quadratic) and the mean time between stepping bouts – with the SPPB score.**

| Model terms | B coefficient [95%CI] | Z | p |
| --- | --- | --- | --- |
| Data from two visits  (observations = 2008, n = 597) |  |  |  |
| Mean time between stepping bouts (mins) | -0.046 [-0.066 to -0.027] | -4.67 | <0.001 |
| Slower-paced steps (1000 steps) | -0.067 [-0.119 to -0.015] | -2.51 | 0.012 |
| Faster-paced walking steps (1000 steps) | 0.516 [0.353 to 0.678] | 6.23 | <0.001 |
| Faster-paced walking steps [squared] (1000^2^ steps) | -0.051 [-0.077 to -0.024] | -3.76 | <0.001 |
| Data from three visits  (observations = 1834, n = 510) |  |  |  |
| Mean time between stepping bouts (mins) | -0.045 [-0.066 to -0.024] | -4.17 | <0.001 |
| Slower-paced steps (1000 steps) | -0.053 [-0.108 to 0.003] | -1.87 | 0.062 |
| Faster-paced walking steps (1000 steps) | 0.509 [0.340 to 0.678] | 5.91 | <0.001 |
| Faster-paced walking steps [squared] (1000^2^ steps) | -0.052 [-0.079 to -0.025] | -3.74 | <0.001 |
| Data from four visits  (observations = 1216, n = 304) |  |  |  |
| Mean time between stepping bouts (mins) | -0.032 [-0.059 to -0.005] | -2.33 | 0.020 |
| Slower-paced steps (1000 steps) | -0.025 [-0.091 to 0.040] | -0.76 | 0.445 |
| Faster-paced walking steps (1000 steps) | 0.378 [0.181 to 0.575] | 3.76 | <0.001 |
| Faster-paced walking steps [squared] (1000^2^ steps) | -0.036 [-0.065 to -0.006] | -2.35 | 0.019 |
